# Excited catatonia in a patient with fatal pulmonary embolism and a successful treatment strategy

**DOI:** 10.1186/s12888-018-1925-2

**Published:** 2018-10-19

**Authors:** Wei-Ting Tseng, Tiao-Lai Huang

**Affiliations:** Department of Psychiatry, Kaohsiung Chang Gung Memorial Hospital and Chang Gung University College of Medicine, No. 123, Dapi Road, Niaosong District, Kaohsiung City, 833 Taiwan

**Keywords:** Catatonia, Schizoaffective disorder, Cardiac arrest, Pulmonary embolism, Benzodiazepine

## Abstract

**Background:**

Patients with psychiatric disorders in critical condition are difficult to treat. In this study, we report on a patient with underlying schizoaffective disorder who developed catatonia, cardiac arrest, and pulmonary embolism, as well as a successful treatment strategy.

**Case presentation:**

The inpatient is a 41-year-old morbidly obese male with schizoaffective disorder whose clozapine dosage was titrated from 100 mg to 175 mg due to auditory hallucination and agitation. The patient abruptly developed acute cardiopulmonary symptoms associated with an elevated troponin-I level. He was transferred to a cardiac intensive care unit, where he remained for 3 days. He was also found to have excited catatonic symptoms, and the lorazepam-diazepam protocol was initiated to quickly relieve the catatonia. Once the coronary angiogram was read as normal, the patient was transferred back to the psychiatric ward. However, the patient then suffered from in-hospital cardiac arrest. He was resuscitated and again transferred to the medical intensive care unit. Computed tomography confirmed the diagnosis of a pulmonary embolism. The patient was treated with Rivaroxaban 30 mg/d for the first 21 days, followed by 20 mg daily for 3 months. To control his severe and refractory psychotic symptoms, the patient was re-prescribed clozapine. During the 15-month follow-up period, the patient demonstrated a fair response and tolerability to clozapine 150 mg without symptoms relapse and no thromboembolic event.

**Conclusion:**

This report can serve to remind psychiatrists and physicians to be aware of fatal conditions in patients with psychiatric diseases and physical illnesses.

## Background

Previous studies have reported that antipsychotic (AP) drugs may increase the risk of developing pulmonary embolism (PE), especially clozapine and low-potency first-generation APs [1]. A potentially fatal and complicated medical emergency in patients with psychiatric disease, pulmonary embolisms are frequently missed at first, resulting in a high mortality rate. Early detection and aggressive management are important in patients with psychiatric diseases, catatonia, and critical physical problems.

In this report, we present a 41-year-old male inpatient with schizoaffective disorder that developed acute cardiopulmonary symptoms and catatonic symptoms. The patient had two intensive care unit (ICU) stays, with an intra-hospital cardiac arrest in between them. A pulmonary embolism was eventually confirmed. After intensive treatments for PE and a benzodiazepine prescription to relieve the catatonia, he had a full recovery. The patient continued to be well during the 15-month follow-up, despite re-introducing clozapine.

## Case presentation

Mr. T, a 41-year-old Taiwanese man, with educational level of 12 years, suffering from schizoaffective disorder for more than 10 years, ever hospitalized to psychiatric wards for 7 times due to disease relapses, despite of more than 2 antipsychotic trials. Before this episode, he had been treated with clozapine 100 mg and lithium 900 mg daily with fair compliance and no remarkable side effects for 2 years. He was obese (body mass index, 39 kg/m^2^), but a lifelong non-smoker with no alcohol or substance abuse, no systemic disease in his medical history and no other comorbid psychiatric disease like personality disorder. He lived with his parents, was unemployed, and was capable of doing simple housework at home.

One day, the patient was sent to the emergency room of a medical center because he had been hearing voices for more than one week. Initial examinations indicated stable vital signs and normal laboratory results, electrocardiogram, and plain chest film. The patient was admitted to the psychiatric unit.

After admission, he was observed to be talking to himself and to have an elevated mood, auditory hallucinations of commanding voices, referential and persecutory delusions, and the hyperactive and agitated behaviors of squatting, standing up, crawling on the ground, and violently attacking others. Some excited catatonic symptoms were also noted, including restlessness, stereotypy, impulsivity, and combativeness. With regard to controlling his agitation and violence symptoms, he was managed with protective restraints and injected lorazepam 2 mg 1 ampule and haloperidol 5 mg 1 ampule intramuscularly, a total of six doses in the first week. A primary regimen of lithium 900 mg/d and clozapine 100 mg/d was used and titrated up to 175 mg/d. Two days after clozapine titration, the patient developed acute cardiopulmonary symptoms, including cold sweats, chest tightness, orthopnea, and tachycardia. Lab results showed elevated cardiac enzymes of CK-MB 37.7 ng/mL, troponin I 3.328 ng/mL, increased WBC 16500/mm^3^, and increased creatine kinase 8045 U/L with 100% MM form. Electrocardiogram showed sinus tachycardia and ST-segment depression in V1–V4. Due to suspected acute non-ST elevation myocardial infarction, the patient was transferred to the cardiac care unit (CCU), where he was managed with enoxaparin subcutaneously and dual antiplatelet agents (DAPT), and the antipsychotics and lithium were discontinued.

His catatonia symptoms persisted, and the patient scored a 20 on the Bush–Francis Catatonia Rating Scale (BFCRS) [2]. A diazepam intravenous drip (10 mg/500 ml infused in normal saline at a rate of 1.25 mg/h) and oral-form lorazepam 4 mg/d were administered [3]. With this treatment, the BFCRS score became 0. After staying 3 days in the CCU, the coronary angiography was reported as being normal, and the enoxaparin and DAPT were discontinued. With a relatively stable physical condition, the patient was transferred back to the psychiatric unit.

However, 3 days after discontinuing enoxaparin and DAPT, the patient suddenly collapsed after defecating in the psychiatric ward. He was resuscitated for 5 min, regaining his pulse, and presented with hypoxia with 80% saturation under Ambu bagging. Laboratory testing showed an elevated d-dimer level > 35 mg/L FEU. The electrocardiogram showed sinus tachycardia and ST depressed at precordial leads V1 -V4. The patient was immediately intubated and once again transferred to the medical ICU. During the second ICU stay, which lasted 7 days, the patient gradually improved and was extubated. While the chest computed tomography reported multifocal filling defects in the bilateral pulmonary arteries, which is in line with a pulmonary embolization, oral rivaroxaban 30 mg/d was given, and the patient was sent back to the psychiatric ward. Since the patient presented with refractory psychotic symptoms and severely agitated behaviors, which may have disturbed his physical recovery, clozapine was prescribed again with an initial dose of 50 mg/d, and the dosage was carefully titrated up to 150 mg as of his discharge approximately 2 weeks later, when he fully recovered physically and mentally. The patient was managed with rivaroxaban 30 mg/d for the first 2 weeks and then 20 mg/d for 3 months.

During the outpatient follow-up period of 15 months, at which point this report was prepared, the patient remained free of any further psychotic, mood, or thromboembolic episodes.

## Discussion

Timely identifying and relieving catatonia in physically ill patients is crucial, as delayed treatment may lead to a prolonged diagnosis and then recovery of underlying physical problems or serious complications, like venous thromboembolism (VTE) [4, 5]. Catatonia is considered a risk factor of deep vein thrombosis, which has a higher incidence rate of 25.3% in catatonic patients, particularly in patients with the retarded form of catatonia rather than the excited form of catatonia [6]. With regard to treating catatonia, the lorazepam-diazepam protocol has been reported to be a rapid and effective strategy for relieving catatonia in patients with schizophrenia, mood disorders, and general medical conditions [3, 7–9]. In this case, we noticed the excited catatonia episode when the patient was sent to the CCU and prescribed a continuous diazepam intravenous drip and oral-form lorazepam 4 mg/d. The benzodiazepine rapidly relieved the patient’s catatonic symptoms so that he could be properly engaged in the intensive medical treatments.

PE, which is among the most life threatening medical emergencies, occurs when clotted blood enters the pulmonary arterial circulation [10]. Patients with similar comorbidities and clot burdens may experience drastically different clinical presentations, ranging from none to sudden death. A retrospective observation study reported that approximately one-third (33.5%) of patients who presented to the emergency department with an acute PE were undiagnosed before being sent home or hospitalized [11].

The risk factors of PE have been well documented in previous studies and include such factors as obesity, pregnancy, malignancy, thrombophilia, recent surgery or major trauma, immobility, smoking, and the use of estrogen-containing medications. Recently, atypical APs such as clozapine ware also reported to be associated with mortality due to PE. The estimated rate of PE in patients treated with clozapine is about 27.5 times higher than in the general population (95% CI 17.1–42.2) [12]. Other evidence has indicated that PE should be suspected in any APs users, even if recently discontinued or decreasing the dose, and particularly with any comorbid risks of thromboembolism, such as immobility [13].

The bio-mechanism of the relationship between clozapine use and PE remains largely unknown, but several hypotheses have been proposed, such as drug induced sedation, obesity, increased levels of antiphospholipid antibodies, hyperhomocysteinemia, hyperprolactinemia, aggregation of platelets antibodies and venous stasis exacerbated by sedation, and immobility secondary to sedation or restraint [1, 13–15]. Based on this patient’s clinical course, in addition to his clozapine use, other risk factors may have also contributed to the PE, including obesity, temporary immobility under restraints, and catatonia symptoms. The patient had no smoking history, immobility or bed rest for more than 72 h, previous thromboembolism history, or family history of thromboembolism. Wells’ score for PE on the cardiac arrest episode was 4.5, which was among the moderate risk group (27.8%) [16].

## Conclusion

In this report, we present a complicated patient with underlying schizoaffective disorder that developed excited catatonia symptoms and cardiac arrest and was diagnosed with a pulmonary embolism. We further provided a successful management strategy with use of lorazepam-diazepam protocol for such critical conditions. We hope that this report will serve as a reminder to psychiatrists and physicians to be aware of fatal conditions in patients with psychiatric diseases and medical illnesses.

## Abbreviations

AP: antipsychotic
BFCRS: Bush–Francis Catatonia Rating Scale
CCU: cardiac care unit
DAPT: dual antiplatelet agents
ICU: intensive care unit
PE: pulmonary embolism
VTE: venous thromboembolism
